# Tradition as a Stepping Stone for a Microbial Defined Water Kefir Fermentation Process: Insights in Cell Growth, Bioflavoring, and Sensory Perception

**DOI:** 10.3389/fmicb.2021.732019

**Published:** 2021-11-04

**Authors:** Sarah Köhler, Maximilian Schmacht, Aktino H. L. Troubounis, Marie Ludszuweit, Nils Rettberg, Martin Senz

**Affiliations:** ^1^Department Bioprocess Engineering and Applied Microbiology, Research and Teaching Institute for Brewing (VLB) in Berlin, Berlin, Germany; ^2^Technische Universität Berlin, Faculty III Process Sciences, Chair of Bioprocess Engineering, Institute of Biotechnology, Berlin, Germany; ^3^Research Institute for Beer and Beverage Analysis, Research and Teaching Institute for Brewing (VLB) in Berlin, Berlin, Germany

**Keywords:** water kefir, co-culture, lactic acid bacteria (LAB), yeast, bioflavoring, process development, yeast extract

## Abstract

A process development from a traditional grain-based fermentation to a defined water kefir fermentation using a co-culture of one lactic acid bacterium and one yeast was elaborated as a prerequisite for an industrially scalable, controllable, and reproducible process. Further, to meet a healthy lifestyle, a low ethanol-containing product was aimed for. Five microbial strains—*Hanseniaspora valbyensis*, *Dekkera bruxellensis*, *Saccharomyces cerevisiae*, *Liquorilactobacillus nagelii*, and *Leuconostoc mesenteroides*—were used in pairs in order to examine their influence on the fermentation progress and the properties of the resulting water kefir products against grains as a control. Thereby, the combination of *H. valbyensis* and *L. mesenteroides* provided the best-rated water kefir beverage in terms of taste and low ethanol concentrations at the same time. As a further contribution to harmonization and reduction of complexity, the usage of dried figs in the medium was replaced by fig syrup, which could have been proven as an adequate substitute. However, nutritional limitations were faced afterward, and thus, an appropriate supplementation strategy for yeast extract was established. Finally, comparative trials in 5-L scale applying grains as well as a defined microbial consortium showed both water kefir beverages characterized by a pH of 3.14, and lactic acid and aromatic sensory properties. The product resulting from co-culturing outperformed the grain-based one, as the ethanol level was considerably lower in favor of an increased amount of lactic acid. The possibility of achieving a water kefir product by using only two species shows high potential for further detailed research of microbial interactions and thus functionality of water kefir.

## Introduction

Traditionally fermented beverages enjoy increasing popularity against the background of a conscious and healthy lifestyle (Kandylis et al., 2016). Especially sour fermented beverages, such as kombucha or kefir, promise health benefits, e.g., antimicrobial, anticancer, or probiotic, by their indigenous microorganisms (Prado et al., 2015; Tamang et al., 2016; Ghosh et al., 2019). Thereby, water kefir (WK), which is based on sugary water (usually sucrose is used) and a fruit component, offers a vegan and lactose-free alternative. The characteristic microorganisms of WK are lactic acid bacteria (LAB) and different yeasts, but also acetic acid bacteria can be involved (Bourrie et al., 2016). The microorganisms are organized in so-called grains that are based on exopolysaccharides mainly produced by the LAB (Nielsen et al., 2014).

Generally, the so-called cross-feeding occurs when LAB and yeast are fermented in co-culture meaning that both groups of organisms can profit from the metabolic products of one another (Bader et al., 2010; Smid and Lacroix, 2013; Stadie et al., 2013; Zhang et al., 2017). This is especially important in the case of WK, where the nutrients in general and nitrogen in particular are limited and may induce sulfurous off-flavors (Vardjan et al., 2013; Laureys et al., 2018). Lactobacilli produce lactate in the course of their carbohydrate metabolism, which yeast can absorb and break down. This prevents a sharp drop in pH and aids the lactobacilli in continuing their usual metabolic activities. The yeasts in turn produce a large number of amino acids and also some vitamins, such as vitamin B6 (Stadie et al., 2013), which are essential nutrients for lactobacilli (Ponomarova et al., 2017; Zhang et al., 2017). Due to this interaction, stimulated growth of lactobacilli is often observed in a co-culture compared with the pure culture (Stadie et al., 2013; Vardjan et al., 2013; Bechtner et al., 2019). Further studies describe culture-related differences in the expression of genes that affect the metabolism of carbohydrates and amino acids (Yamasaki-Yashiki et al., 2017; Bechtner et al., 2019), as well as exopolysaccharide formation (Yamasaki-Yashiki et al., 2017) and aggregation factors (Bechtner et al., 2019) involved. In addition, during co-cultivation, the aerobic metabolism of the yeasts reduces the oxygen content of the nutrient medium, which in turn can be beneficial for the growth of lactobacilli (Mendes et al., 2013; Nejati et al., 2020). Although some general modes of interactions between different groups of microorganisms are known (Nejati et al., 2020), the relationship between different strains and whether the interactions are synergistic or counteractive is still not fully understood and remains a topic of current research. Additionally, the mixture of the specifically involved organisms results in a distinct profile of organic acids and exopolysaccharides that have an important influence on the sensory properties of the final beverage (Bader et al., 2010; Bertsch et al., 2019; Jin et al., 2019).

Besides the consortium of microorganisms, also the fruit component is rather complex. Traditionally, e.g., figs, dates, raisins, and/or lemons, were used to produce WK (Açik et al., 2020). However, as with every natural ingredient, quality can vary; and there may occur seasonal effects that influence the industrial production. Moreover, the choice of the fruit component significantly influences the fermentation process by the provision of nutrients for the involved microorganisms as well as the final sensory properties of the beverage.

In summary, industrializing the production of WK with a complex mixture of raw materials and microorganisms is very difficult in terms of final product properties and especially reproducibility (Laureys and de Vuyst, 2017; Nejati et al., 2020). To the knowledge of the authors, successful industry-scale water kefir products are scarce because the management of the fermentation process to obtain reproducible products is similarly complex as the microbial consortia themselves. Therefore, the companies keep their recipes and control strategies guarded. A systematic understanding of mutual interactions aids in the specific control of the production process resulting in safe, defined, and reproducible products. Consequently, efforts to develop defined fermentations applying specific starter cultures are undertaken, whereby a reasoned selection of production strains is of utmost importance. Thereby, one can assume that the level of interactions in a defined co-culture is lower than a complex microbial community, posing the risk of also resulting in a less complex product. However, the application of defined co-cultures has the potential to be better controllable due to its lower level of complexity resulting in less “adjustment screws” and thus lower product deviations.

In order to get a deeper insight which microorganisms originating from natural WK grains contribute synergistically to the fermentation progress resulting in an organoleptic characteristic beverage, systematic experiments to reduce the microbial complexity were conducted. Furthermore, the main focus was the production of a lactic acidic beverage by co-culture fermentation, which should preferably contain a low amount of alcohol meeting the consumer demands for a healthy drink. Thereby, the influence of the available nutrients, such as amino acids and trace elements, on the microbial interactions and the sensory properties represented by different microbially built chemical compounds as well as possible limitations were considered. This paper finally presents a comparison of a traditionally produced complex WK and a fermented beverage product produced with specific starter cultures mimicking WK regarding the fermentation parameters as well as the final product properties.

## Materials and Methods

### Microorganisms

In the present studies, WK grains were used. These have been used successfully for many years for the traditional production of WK via back-slopping processes. The microbial strains used as defined cultures were isolated and identified from these mentioned grains. Therefore, grains were homogenized by using the ULTRA-TURRAX^®^ T25 basic (IKA^®^ -Werke GmbH & CO. KG, Staufen, Germany), and different dilutions of the homogenized liquid were plated on yeast extract (YE) dextrose agar as well as MRS agar according to De Man et al. (1960). Incubation took place at 26, 30, and 37°C under aerobic and anaerobic conditions. Pure cultures were gained by repeating the plating step of single colonies several times. From those single colonies, material was taken for DNA extraction (Phire^TM^ Plant Direct PCR Master Mix, Thermo Fisher Scientific Inc., Waltham, MA, United States) followed by PCR amplification using primers 8(F) (5′-AGAGTTTGATCCTGGCTCAG-3′) and 1492(R) (5′-GGTTACCTTGTTACGACTT-3′) for bacteria (Turner et al., 1999) and ITS1(F) (5′)-TCCGTAGGTGAACCTGCGG-3′) ITS4(R) (5′-TCCTCCGCTTATTGATATGC-3′) for yeast (Op De Beeck et al., 2014) followed by sequencing and blasting the resulting PCR products for species identification. Selected isolates of LAB and yeast were transferred in the VLB strain collection and further examined in fermentation studies: *Hanseniaspora valbyensis* Hs-0302 (Han), *Dekkera bruxellensis* Br-0115 (Dek), *Saccharomyces cerevisiae* Sa-07366 (Sac), *Liquorilactobacillus nagelii* La-3804 (formerly *Lactobacillus nagelii*; Liq), and *Leuconostoc mesenteroides* Le-0304 (Leu). Every yeast strain was combined with every bacterium in co-culture, which led to six applied combinations.

### Media Composition and Preparation

Two different media, herein referred to as basis medium and modified medium, were used for the fermentation studies. The basis medium was established on a common recipe for WK production. In detail, it was composed of the following (per liter): 1 dry fig (Alesto, Lidl Dienstleistung GmbH & Co. KG, Neckarsulm, Germany), 60 g of brown cane sugar (Fairglobe, Lidl Dienstleistung GmbH & Co. KG), and 7.5 mL of lemon juice concentrate (Solevita Zitrone, Lidl Dienstleistung GmbH & Co. KG). The modified medium was composed of the following (per liter): 9 mL of fig syrup (Schoeneberger, Salus Pharma GmbH, Bruckmühl, Germany), 60 g of brown cane sugar, 7.5 mL of lemon juice concentrate, and a defined concentration of either X-SEED KAT YE or X-SEED Peptone (Ohly GmbH, Hamburg, Germany), a yeast peptone. Both yeast derivatives were of food grade quality. For media preparation, sugar, dry fig/fig syrup, and YE when applicable were dissolved in boiling tap water (80% of needed volume). After being cooled down to room temperature (20°C), lemon juice concentrate was added, and batches were filled up to desired volume with lukewarm tap water.

### Experiments

#### Pre-cultivation

The strains were pre-cultured in two steps. In both steps, yeast strains were pre-cultured in 250-mL shaking flask at 130 rpm and 30°C for 72 h in YE dextrose (YED) medium containing 50 g/L of glucose (AppliChem GmbH, Darmstadt, Germany) and 10 g/L of YE (SERVABACTER^®^, SERVA Electrophoresis GmbH, Heidelberg, Germany), pH 5.5. LAB were pre-cultured as stand culture in closed 250-mL bottles at 30°C for 72 h in MRS medium according to De Man et al. (1960) from Difco^TM^ (Becton Dickinson GmbH, Franklin Lakes, NJ, United States). Pre-culture 1 was inoculated with 1% (v/v) of cryo-culture stocks, and pre-culture 2 was inoculated with 1% (v/v) of pre-culture 1. Before the main cultures were inoculated, the microbial suspensions were washed in 0.9% sodium chloride solution to reduce the influence of media components. WK grains were cultivated in a typical matrix [60 g of sucrose (AppliChem GmbH, Darmstadt, Germany), 100 g of pre-washed grains, and two dried figs per liter, 26°C] over several 3-day cycles in the back-slopping process.

#### Testing of Different Combinations of Microbial Strains

In the first part of the studies, fermentations with different combinations of two microbial strains in co-culture were investigated. Thereby, 1 ⋅ 10^6^ yeast cells/mL and 1 ⋅ 10^7^ LAB/mL were applied in co-culture. The ratio yeast/LAB (1:10) was derived from the respective cell concentrations in the supernatant of WK fermented by grains. For comparison, fermentations with WK grains were investigated simultaneously. Thereby, 100 g of WK grains per liter was used for the fermentation. For the investigations, the basis medium, which contained dried fruits, was used. The fermentations were conducted in 1-L glass bottles with loosely sealed caps at 22°C (±2°C) (room temperature) as stand cultures. At the start of the fermentation and before sampling, the samples were mixed by mild horizontal shaking. The sampling was executed once per day for offline analyses, which included determination of cell count, high-performance liquid chromatography (HPLC) analysis, pH measurement, and sensory analysis. After 7 days of fermentation, the products showed the best obtainable characteristics and were analyzed toward a wide range of volatile components (see section “Analyses of Volatile Components”). The fermentation studies were executed in biological duplicates. The most appropriate combination of microorganisms was then applied in further trials.

#### Testing the Impact of Different Yeast Extracts and Concentrations During Fermentations With Leu + Han

In further experiments, the components of the basis medium were adapted to make the composition more defined, further called modified medium. Thereby, the impact of the supplementation of either X-SEED KAT YE or X-SEED Peptone (Ohly GmbH), a yeast peptone, was tested in the concentration of 0.2 and 1.0 g/L each. Roughly, these two yeast supplements differed in their protein content (75% in X-SEED KAT vs. 67% in X-SEED Peptone) and composition [higher proportion of short-chain peptides and amino acids in X-SEED KAT (available at X-SEED product sheets)]. Furthermore, the modified medium contained fig syrup in a defined volume (cf. section “Media Composition and Preparation”) to avoid the usage of whole dried fruits. The sampling was executed once per day for offline analyses, which included determination of cell count, HPLC analysis, pH measurement, and sensory analysis. After 7 days of fermentation, aroma analyses of the final products took place. The fermentation studies were executed in biological duplicates.

#### Five-Liters Fermentation With Water Kefir Grains or the Co-culture of Leu + Han Under Defined Conditions

Finally, fermentations in 5-L bioreactors with WK grains as well as the co-culture combination Leu + Han with the modified medium were performed. For this purpose, 4.5 L of modified medium including 1.3 g/L of X-SEED KAT and 4.5 L of modified medium without YE was used in the case of the co-culture Leu + Han and WK grains, respectively. Fermentations took place in 5-L Biostat^®^ B Twin bioreactors (Sartorius AG, Göttingen, Germany), at 26°C with a low stirring rate of 20 rpm and no aeration. The pH value and dissolved oxygen (pO_2_-%-saturation) were measured online via EASYFERM PLUS VP PH/RX 325 and VISIFERM DO ECS 325 H0 (Hamilton Germany GmbH, Gräfelfing, Germany) probes. The sampling was executed three times a day for offline analyses, which included determination of cell count, HPLC analysis, pH measurement, and once-per-day sensory analysis. Fermentations were carried out in biological triplicates. Data from identical sampling times were shown as the mean value of a triple, double, or single sampling and were marked accordingly.

### Analytical Methods

#### Determination of Total Cell Concentration by Coulter Counter

The total cell concentration was analyzed by using impedance measurement (Multisizer^TM^ 3, Beckman Coulter GmbH, Brea, CA, United States). In the case of samples including grains, only the liquid fraction was analyzed. Ten microliters of the sample was diluted in 10 mL of Isoton II, and 50 μL thereof was analyzed using a 30 μL capillary. With the use of the Multisizer^TM^ 3 Software Version 5.53, the pulse data were converted to size features. In the case of co-culturing, LAB and yeast strains were used, which differed in their cell sizes; and thus, a discrimination of the respective populations was possible. Particles with a size of 0.6–2.0 μm were considered as LAB cells, whereas particles with a size of 2.0–10.0 μm were considered as yeast cells based on cell size distribution in pure cultures, respectively. Their concentration per mL was calculated. A potential overlap of the LAB and yeast populations by crossing cell sizes was in neither case significant and thus was neglected for the determination of the real population distribution ratio, which was a good approximation. For the strains used, a chain formation could not have been microscopically verified in the conditions applied in the experiments.

#### Determination of Low-Molecular Sugars, Ethanol, and Organic Acids

The analyses of low-molecular sugars (in detail glucose, fructose, and sucrose) and ethanol and organic acids (more detailed lactic acid and acetic acid) were conducted via HPLC (Knauer Wissenschaftliche Geräte GmbH, Berlin, Germany) applying 10 μL of sample on a Nucleogel^®^ Ion 300 OA column (Macherey-Nagel GmbH & Co. KG, Düren, Germany) at 40°C column temperature. The separation of the target compounds was achieved using an isocratic elution with 5 mmol/L of H_2_SO_4_ at a flow rate of 0.4 mL/min. The sugar and ethanol detection was performed in a refractive index detector. Organic acids were detected via multiple wavelength detector at a wavelength of 210 nm. The residual sugar was defined as the sum of glucose, fructose, and sucrose.

#### Analyses of Volatile Components

The final fermented products as well as the respective unfermented beverage bases were tested for a number of volatile components and fatty acids, which are described in detail as follows.

##### Quantification of Acetaldehyde, Higher Alcohols, and Acetate Esters

The quantification of acetaldehyde, higher aliphatic and aromatic alcohols, and acetate esters was determined by static headspace gas chromatography with flame ionization detection (HS-GC-FID) according to method 9.39 outlined in the European Brewery Convention (EBC) (Analytica-EBC, 1997). The GC-FID instrument used was a Shimadzu GC-2010 (Shimadzu Corp., Kyôto, Japan) equipped with a DB-Wax (60 m × 0.32 mm × 0.5 μm film thickness) from Agilent (Santa Clara, CA, United States). Quantification of higher alcohols and acetate esters was reached using butan-1-ol and phenol as internal standards added at a concentration of 15 mg/L. The calibration ranges of the compounds differed and were as follows: acetaldehyde (1.3–75 mg/L), ethyl acetate (1.3–75 mg/L), propanol (2.5–75 mg/L), 2-methylpropan-1-ol (isobutanol) (2.5–75 mg/L), 3-methylbutyl acetate (isoamyl acetate) (0.2–10 mg/L), 2-methyl-1-butanol (2.5–75 mg/L), 3-methyl-1-butanol (2.5–100 mg/L), phenyl ethanol (2.5–75 mg/L), and phenylethyl acetate (0.05–5 mg/L). The lowest concentration of the calibration range was defined as limit of quantification for each compound.

##### Determination of Ethyl Esters

Ethyl butanoate, ethyl hexanoate, ethyl octanoate, ethyl decanoate, and ethyl dodecanoate were determined by headspace–solid-phase microextraction (HS-SPME) gas chromatography–mass spectrometry (GC-MS). The GC-MS system consisted of a Shimadzu GC 2010 interfaced with a MS-QP2010 Plus (Shimadzu Corp.) equipped with a Gerstel MPS 2XL auto sampler (Gerstel, Linthicum Heights, MD, United States) for automated HS-SPME sampling. Data evaluation was done using the GCMSsolution software Version 4.45 SP1 (Shimadzu Corp.). Esters were extracted from 2 mL of liquid sample using a 50/30 μm divinylbenzene/carboxen/polydimethylsiloxane (DVB/CAR/PDMS) fiber (Supelco, Bellefonte, PA, United States). The column used for chromatographic separation was a HP-5MS UI column [30 m × 0.25 mm i.d. × 0.25 μm film thickness from Agilent (Santa Clara)]. The extraction and GC parameters were used as described by Dennenlöhr et al. (2020a). The mass spectrometer was operated in selected ion monitoring (SIM) mode, using the following qualifier and quantifier ions: ethyl butanoate (*m*/*z* = 71 and *m*/*z* = 60), ethyl hexanoate (*m*/*z* = 88 and *m*/*z* = 101), ethyl octanoate (*m*/*z* = 88 and *m*/*z* = 127), ethyl decanoate (*m*/*z* = 88 and *m*/*z* = 73), and ethyl dodecanoate (*m*/*z* = 88 and *m*/*z* = 101). The calibration ranges for all ethyl esters were 5–1,000 μg/L. The lowest concentration of the calibration range (5 μg/L) was defined as limit of quantification. Isotopically labeled *d*_5_-ethyl hexanoate (*m*/*z* = 93 and *m*/*z* = 106) was used as internal standard at a concentration of 100 μg/L.

##### Determination of Volatile Sulfur Components

The analyses of volatile sulfur components were accomplished by application of two separate HS-SPME GC-MS/MS methods that were both run on an Agilent Technologies 7890B gas chromatograph interfaced to a 7000C Triple Quadrupole mass spectrometer (Agilent). This GC-MS/MS setup was equipped with a Gerstel MPS 2XL sampler (Gerstel) for automated HS-SPME sampling, and the column used for chromatographic separation was a HP-5ms Ultra Inert GC Column (30 m × 0.25 mm, × 0.25 μm film thickness from Agilent). Agilent MassHunter Workstation Software—Qualitative Analyzes (ver. B.07.00) was used for data analyses. Method 1 covered methanethiol, ethanethiol, propane-1-thiol, and butane-1-thiol. The second method covered the thioesters and sulfides, namely, *S*-methyl thioacetate, ethyl thioacetate, carbon disulfide, diethyl sulfide, dimethyl disulfide, and dimethyl trisulfide. Sample preparation for the analyses of volatile thiols was done using the on-fiber derivatization (OFD) assay as published by Dennenlöhr et al. (2020b). The conditions during sample preparation and the GC separation were also as previously published. The mass spectrometer was operated in electron impact ionization (70 eV) multiple-reaction ion monitoring (MRM) mode using the following transitions for quantification and qualification: methanethiol (*m*/*z* = 228 → 181 and *m*/*z* = 181 → 161), ethanethiol (*m*/*z* = 242 → 181 and *m*/*z* = 213 → 45), propane-1-thiol (*m*/*z* = 256 → 181 and *m*/*z* = 214 → 181), and butane-1-thiol (*m*/*z* = 270 → 89 and *m*/*z* = 213 → 181). Analytes were calibrated in a range from 5 to 500 ng/L (methyl mercaptan: 100–10,000 ng/L) and the lowest concentration of the calibration range (5 ng/L for ethanethiol, propane-1-thiol, and butane-1-thiol and 100 ng/L for methanethiol) was defined as limit of quantification. 1-Hexanethiol was used as an internal standard (*m*/*z* = 117 → 83 and *m*/*z* = 298 → 117), and it was added to the samples at a concentration of 100 ng/L at the very beginning of the sample preparation. Sample preparation for the analysis of thioesters and sulfides was done under similar conditions as described for the thiols, while the OFD step was skipped. The GC temperature program used to separate the components of interest was as follows: start at 30°C, ramp with 2°C/min to 41°C, then ramp with 40–140°C, then ramp with 60–300°C. The mass spectrometer was operated in MRM except for carbon disulfide that was analyzed in SIM with selected ions *m*/*z* = 76 (quantification) and *m*/*z* = 78 (qualification). The MRM transitions used for quantification and qualification of the remaining components were *S*-methyl thioacetate (*m*/*z* = 90 → 43, no qualifier transition), ethyl thioacetate (*m*/*z* = 104 → 43 and 43 → 42), diethyl sulfide (*m*/*z* = 90 → 62 and 75 → 47), dimethyl disulfide (*m*/*z* = 122 → 94 and 94 → 66), and dimethyl trisulfide (*m*/*z* = 126 → 61 and 79 → 45). Isotopically labeled *d*_6_-dimethyl trisulfide (*m*/*z* = 132 → 82 and 114 → 50) was used as an internal standard and was added to the samples at a concentration of 1 μg/L at the very beginning of the sample preparation. The calibration ranges of the components differed and were as follows: carbon disulfide (0.005–0.1 μg/L), *S*-methyl thioacetate (0.025–20 μg/L), ethyl thioacetate (0.005–4 μg/L), diethyl sulfide (0.005–2 μg/L), dimethyl disulfide (0.005–4 μg/L), and dimethyl trisulfide (0.005–1 μg/L). The lowest concentration of the calibration range was defined as limit of quantification for each component.

##### Quantification of Short- and Medium-Chain Fatty Acids

Short- and medium-chain fatty acids, herein referred to as fatty acids, were determined by HS-SPME-GC-FID. The GC-FID system used was a Shimadzu GC-2010 (Shimadzu Corp.) equipped with an Agilent CP Wax 58 FFAP (50 m × 0.32 mm × 0.32 μm). HS-SPME sampling was done using a Gerstel MPS 2XL auto sampler (Gerstel) equipped with an 85 μm polyacrylate fiber (Supelco). To extract the fatty acids, 2 mL of aliquots of the liquid samples was acidified by addition of 80 μL of 1 M HCl in 10-mL amber headspace vials. To enrich components in the headspace above the sample, sealed headspace vials we incubated for 15 min at 50°C (500 rpm), followed by an extraction for 15 min at 50°C. The loaded fiber was then desorbed for 1 min at 250°C using a split ratio of 2. A temperature program starting at 60°C, followed by a ramp of 17°C/min to 150°C, and followed by a ramp of 8°C/min to 220°C was used to separate the target analytes. The calibration ranges were as follows: butanoic acid (0.15–12 mg/L), 3-methyl butanoic acid (0.05–4 mg/L), pentanoic acid (0.03–2.4 mg/L), hexanoic acid (0.05–4 mg/L), octanoic acid (0.1–8 mg/L), decanoic acid (0.04–3.2 mg/L), and dodecanoic acid (0.03–2.4 mg/L). The lowest concentration of the calibration range was defined as limit of quantification for each component. 4-Methyl pentanoic acid was used as internal standard; it was added to the samples at a concentration of 3 mg/L prior to acidification with HCl.

#### Sensory Analyses

Three trained experts in tasting of sour fermented beverages analyzed sensory properties of the fermentation products. For this purpose, a descriptive and evaluative analysis scheme for sour fermented beverages established at the VLB was used. After sampling, all beverages were stored in closed screw-cap tubes for a maximum of 2 days at 4°C and brought to room temperature shortly before tasting. Samples were tasted in a non-blinded manner.

## Results

In the following, the fermentation progress of the trials examining different microbial combinations (section “Different Lactic Acid Bacteria–Yeast Combinations for Producing Water Kefir Beverages”) as well as adapted media (sections “Using Fig Syrup Compared With Dry Figs Based on Fermentation With Water Kefir Grains” and “Effect of Different Yeast Extracts and Concentrations on the Performance of Leu + Han During Water Kefir Fermentation”) aiming for a more defined WK production process is shown. Finally, 5-L bioreactor fermentations applying the appropriate microorganism combination compared with fermentations with original WK grains were performed (section “Five-Liters Bioreactor Fermentation With Water Kefir Grains and Defined Co-culture Leu + Han”).

### Different Lactic Acid Bacteria–Yeast Combinations for Producing Water Kefir Beverages

Firstly, systematic experiments to reduce the microbial complexity were conducted. Therefore, combinations of one lactic acid bacterium and one yeast strain were applied in basis medium against the usage of grains as a control. The focus was on the production of a lactic acidic beverage, which should preferably contain a low amount of alcohol besides an overall balanced sensory perception.

**FIGURE 1:**
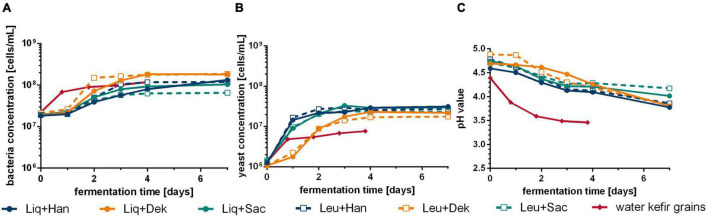
Course of bacteria **(A)** and yeast **(B)** cell concentration and pH change **(C)** during WK fermentation with different starter culture combinations. Data are the mean value of biological duplicates (LAB–yeast combinations) or triplicates (grains). WK, water kefir; LAB, lactic acid bacteria.

**FIGURE 2:**
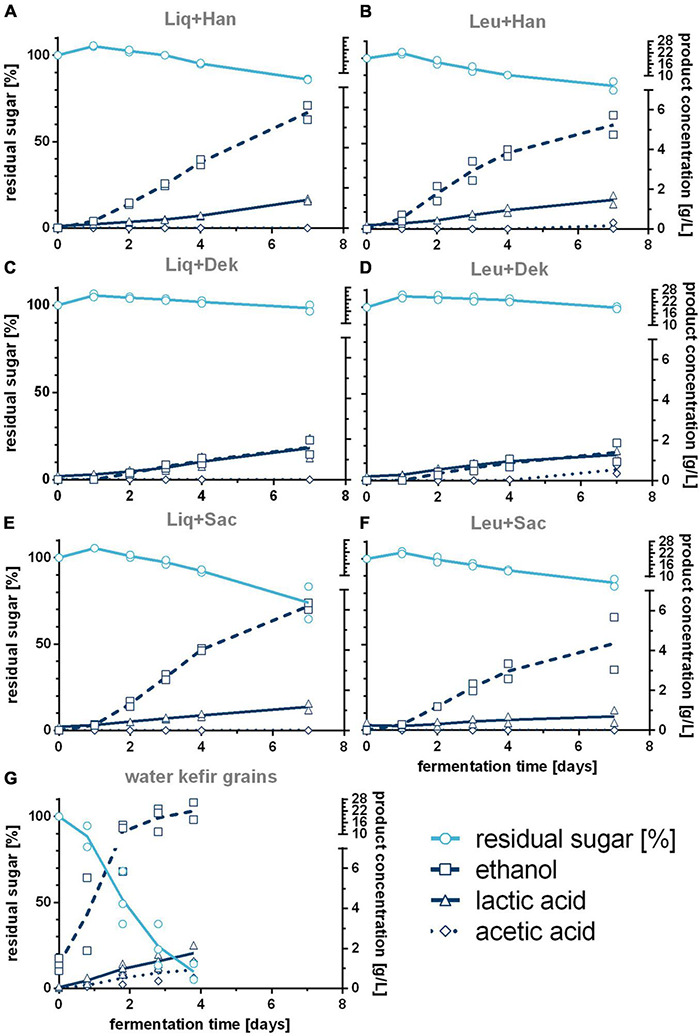
Course of produced ethanol, lactic, and acetic acid and consumed sugar during the WK fermentations with different LAB–yeast combinations **(A–F)** and complex grains **(G)**. Shown are the data of biological duplicates (LAB–yeast combinations) or biological triplicates (complex grains) and the corresponding trend lines. WK, water kefir; LAB, lactic acid bacteria.

**FIGURE 3:**
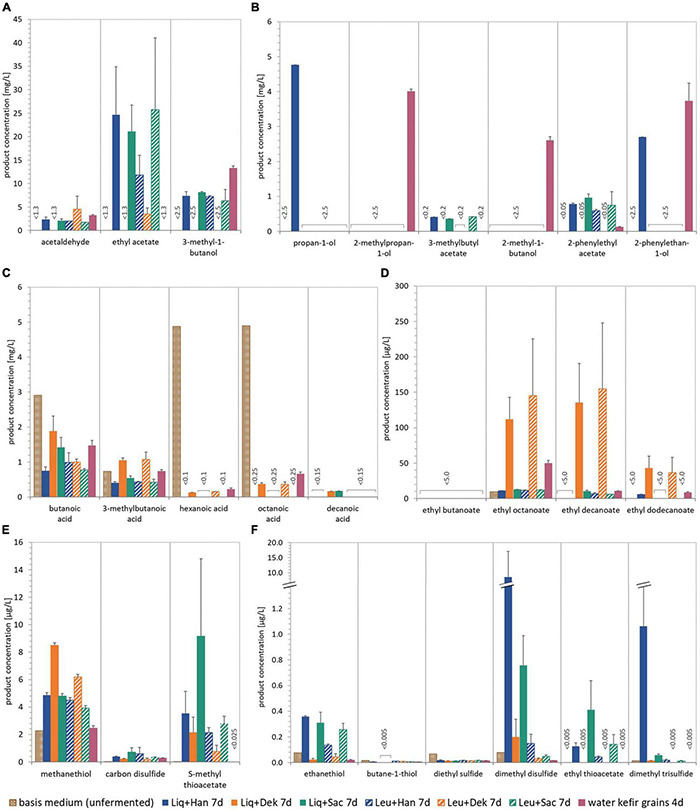
Concentrations of different selected volatile components in the unfermented basis medium and beverages produced with different LAB–yeast combinations and WK grains after 7 and 4 days of fermentation, respectively. The quantified aroma-relevant analytes included higher aliphatic alcohols and esters **(A,B)**, fatty acids **(C)**, fatty acid ethyl esters **(D)**, and thiols and other sulfur components **(E,F)**. The detection limit is specified for the components for which no detection was possible. The analytes pentanoic acid, dodecanoic acid, ethyl hexanoate, and propane-1-thiol (all not shown) were not detected in any sample. Data of the fermented products are the mean value of biological duplicates ± mean deviation. WK, water kefir; LAB, lactic acid bacteria.

Figure 1 demonstrates the differences of cell growth and pH changes during 7 days of fermentation caused by different microorganism combinations. Firstly, the usage of WK grains led to different courses in pH and the concentrations of cells in the liquid phase, metabolites, and volatile components compared with defined combinations (cf. Figures 2, 3). After at least 4 days, the residual sugar was close to zero, and the alcohol content increased to a mean value of finally 24 g/L (3.0 Vol.-%) by the usage of grains, which is shown in Figure 2. In mean, 2 g/L of lactic acid and 1 g/L of acetic acid were produced. The acid concentrations were higher than those produced by the co-cultures. This led to the fastest pH decrease resulting in a final pH value of 3.5 displayed in Figure 1C.

The grains showed the fastest rise of bacterial cell growth compared with the defined co-cultured bacteria cells (1 vs. 2 days to end exponential growth phase). However, at the end of the fermentation, similar bacterial cell concentrations were reached (∼1 ⋅ 10^8^ cells/mL). In contrast, lower final yeast cell counts of 7.64 ⋅ 10^6^ cells/mL than in co-culture (∼2.5 ⋅ 10^7^ cells/mL on average), which showed similar progress of the yeast cell counts, were observed. In the co-culture, the bacteria cell growth depended on the combined yeast. Consequently, the combinations including Dek showed the lowest yeast cell growth compared with the other combinations but the fastest bacteria growth in case of co-culture, which resulted in an amount of 1.8 g/L of lactic acid (cf. Figure 2) and led to low pH values of 3.81. In conjunction with a low sugar consumption (1.2% observed sugar reduction), a low amount of ethanol was produced during the fermentation processes including Dek (<2.0 g/L; ∼0.25 Vol.-%). The low cell growth of Dek in the beginning allowed a faster growth of the LAB. These observations indicate an interaction between the used LAB and Dek regarding the competition for nutrients and differ from the interaction of other used yeasts, Han and Sac.

**FIGURE 4:**
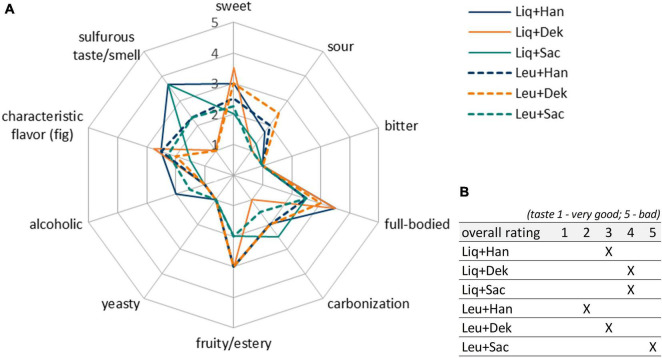
Sensory properties of products fermented with different LAB–yeast combinations after 7 days of fermentation. Shown are the mean data of two samples produced in biological duplicates. The rating scale of the descriptive analysis **(A)** ranges from 1 (imperceptible) to 5 (very pronounced). The overall rating of the taste **(B)** ranges from 1 (very good) to 5 (bad taste). LAB, lactic acid bacteria.

Combinations with Han and Sac led to ethanol concentrations of ∼6 g/L (∼0.76 Vol.-%), which was sensory recognizable in the case of Liq + Han. The combination of Leu + Sac showed the lowest pH decrease to finally 4.3 (cf. Figure 1). This is in accordance with the lowest observed acid production of ∼1 g/L of lactic acid (cf. Figure 2), and the sensory evaluation, in which only a weak acid was perceptible (cf. Figure 4). Figure 4 demonstrates less pronounced typical WK-like sensory attributes—low sourness, carbonization (in case of combinations with Leu), and fruitiness for instance. Sac seemed to have been inhibiting or have less promoting effects on Leu, which could be concluded from the lower bacteria cell concentration (6.57 ⋅ 10^7^ cells/mL) compared with that of the other co-cultures (>1 ⋅ 10^8^ cells/mL) and the resulting lower acidity due to less produced lactic acid.

Figure 3 shows the concentration of different volatile components in the unfermented basis medium as well as the final fermented WK products after 7 days. The unfermented basis medium containing dried figs, sugar, and lemon juice concentrate contained a few volatile components in a detectable range. These were smaller fatty acids as well as low amounts of methanethiol and related sulfurous components (cf. Figures 3C,E,F). In the fermented products, concentrations of the volatile components were generally higher but varied from product to product. The volatile profile of WK fermented by usage of grains differed from that observed for WK from co-cultures. Within the WK resulting from co-cultures, volatile profile differed, and this is attributed to the combination of different microorganisms. Ethyl acetate was the most dominant form of volatile component and was detected in the products containing the yeasts Han and Sac in combination with Liq and Leu, respectively, but did not occur in a detectable range in the product using grains. The detected amounts of higher aliphatic alcohols and esters differed depending on the used combination (cf. Figures 3A,B). It is conspicuous, that either grains or microbial co-cultures produced certain components of these higher aliphatic alcohols and esters. The combination of Dek + Liq as well as Dek + Leu led to no formation of those alcohols. It is of particular note that the latter combination led to the highest amount of fatty acid ethyl esters, which led to the fruity characteristics of the products and which were produced in a much lower quantity by the other combinations. Figure 3C displays a much lower amount of fatty acids, especially hexanoic acid as well as octanoic acid, in the fermented products after 7 days compared with the unfermented basis medium. These components have been metabolized by the cells. Although the components butanoic acid and 3-methylbutanoic acid are the most dominant fatty acids, their concentration was either lower than or the same as concentration in the medium.

In contrast to fruity aroma components, thiols and sulfurous components were found in a detectable range in all combinations as well as in the basis medium, which is displayed in Figures 3E,F. Thereby, the most abundant component was methanethiol, which acts as a precursor for further sulfurous components such as dimethyl disulfide and dimethyl trisulfide (Kinzurik et al., 2020), and was formed by the microbial combination consisting of Dek + Liq as well as Dek + Leu mostly. Other sulfurous components were produced by the usage of Liq in combination with the yeast Han as well as Sac in the highest amount, e.g., ethanethiol as well as dimethyl disulfide. The specific aroma profile of the products led to a specific sensory taste as well, which is demonstrated in Figure 4.

Although the combinations of Dek with Liq and Leu (orange lines in Figure 4) showed a high potential in the expression of aimed WK-like attributes, the usage of these combinations was evaluated as not appropriate for beverage production. After 7 days, the final products were lactic and citric acidic, full-bodied, mildly fruity, and having a dominant fig flavor. However, these products showed a strong off-flavor, which was describable as “mustily,” “stuffy,” and “stale” (not shown in the sensory panel). This can be attributed to the applied yeast, Dek.

Further, the blue lines in Figure 4 display the well-balanced composition between sourness and sweetness of the final products fermented by Han + LAB after 7 days. These results determined the final fermentation duration of 7 days. In contrast, the fermented products by using the yeast Sac showed both lactic and acetic acid taste represented by green lines in Figure 4. Apart from the desired product properties, a highly pronounced sulfurous taste and smell occurred during fermentation, especially in combinations with Liq. The products smelled and tasted like rotten eggs. The most promising products were fermented by the yeast Han. Thereby, the combination with Liq showed stronger alcoholic and sulfurous taste as well as less sourness than in combination with Leu.

Summarizing, the fermentations with the co-cultivation of LAB and yeasts lasted longer to get a final product than the fermentations with grains. The fermentation with WK grains was faster and led to a more complex taste and a higher diversity in perceptible sensory properties but resulted in a higher content of alcohol in the final product. By using defined starter cultures in a co-cultivation, the formation of ethanol was lower and might be more controllable than by fermentations with grains.

Sensory-wise, the combination of Leu + Han was preferred by the panelists, which was based on a well-balanced characteristic between sourness (lactic acid) and sweetness and the presence of desired attributes such as fruity and carbonized. Based on the results, the combination was chosen for further trials.

### Using Fig Syrup Compared With Dry Figs Based on Fermentation With Water Kefir Grains

In order to implement more defined media components in the production process, investigations with fig syrup were executed. For this purpose, different volumes of fig syrup were tested to get the most appropriate concentration equal to the usage of one dry fig per liter. In a test row, basis media were investigated, which differed in the volumes of liquid syrup. Sugar and lemon juice concentrate, as was used above, were added, and sensorial tests were performed. In conclusion, 9.0 mL of fig syrup per liter corresponded to the usage of one dry fig per liter regarding the taste as the basis. Based on this, fermentations with WK grains with the chosen amount of fig syrup compared with the usage of dry figs were performed, and results can be seen in Supplementary Figure 1. In this context, the fermentations showed similar behavior in cell growth, pH value decrease, and metabolic activity as well as sensory properties. Thus, fig syrup was used in following studies.

### Effect of Different Yeast Extracts and Concentrations on the Performance of Leu + Han During Water Kefir Fermentation

**FIGURE 5:**
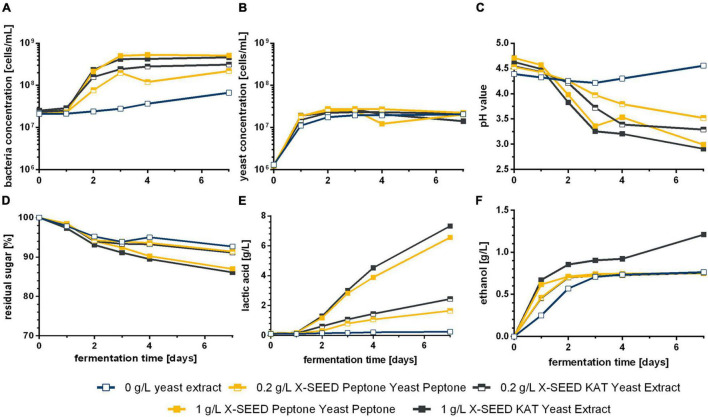
Course of cell concentrations **(A,B)**, pH value **(C)**, residual sugar **(D)**, produced lactic acid **(E)**, and ethanol **(F)** during the WK fermentation by Leu + Han with different types and concentrations of supplemented YE. Data are the mean value of biological duplicates. WK, water kefir; YE, yeast extract.

In order to avoid the noticeable presence of sulfurous components in the final product when applying defined starter cultures, two different YEs, developed for LAB cultivation especially, were used as a sufficient supplement of nutrients. The fermentation performance of the microorganism combination Leu + Han without the supplementation of YE was not as good as in the first study (cf. Figures 1, 2). This might be because of the replacement of dry fig (used in experiments shown in Figure 1) with fig syrup (experiments shown in Figure 5) and an associated lack of nutrients. It was concluded that fig syrup was not sufficient as the only source of nutrition in the case of the here-applied co-cultivation for the WK production. On the contrary, a positive effect of adding YE to the medium for the WK production with two microbial strains was apparent, which was demonstrated by a higher concentration of LAB compared with the fermentation without YE (Figure 5A). Thereby, increasing the YE concentration led to a faster growth of LAB. Accordingly, Figure 5C shows a faster decrease of the pH based on the added YE, especially 1 g/L of X-SEED KAT. Furthermore, the final pH was lower than the values observed during the fermentation with grains. These high pH decreases are because of the high yield of lactic acid concentrations and can be seen in Figure 5E. Lactic acid reached values higher than 7 g/L using 1 g/L of YE, whereas the application of 0.2 g/L of YE led to ∼2 g/L. Concluding, the amount of produced lactic acid differed between different applied concentrations of YE. In contrast, the yeast cell growth was not influenced by different applied YEs and their concentrations (Figure 5B); thus, the yielded ethanol concentration differed remarkably after 3 days of fermentation except for the usage of 1 g/L of X-SEED KAT YE with a slightly higher concentration (Figure 5F).

**FIGURE 6:**
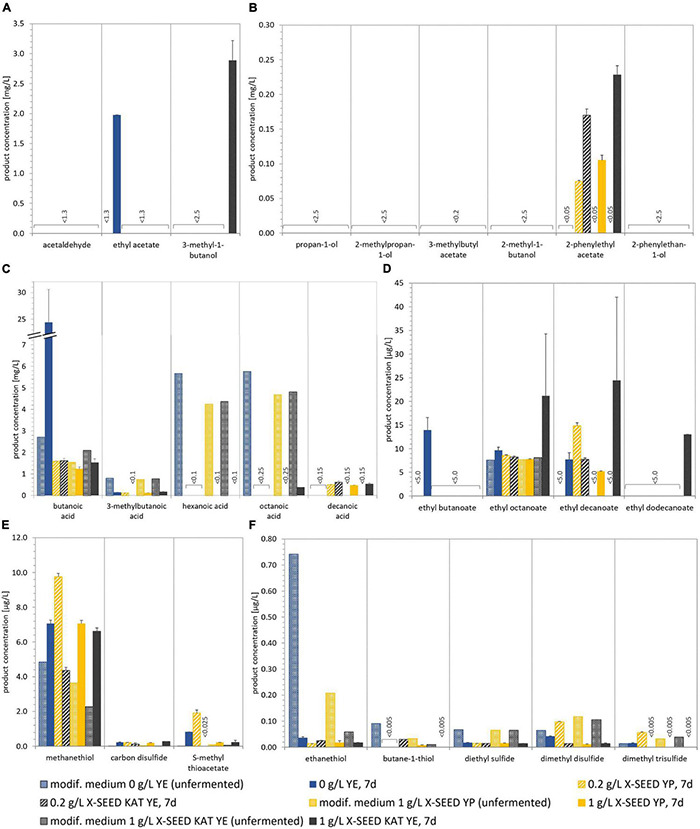
Concentrations of different volatile components in modified media (references) and in 7-day fermented beverages produced with the Leu + Han combination and different types and amounts of YE. The quantified aroma-relevant analytes included higher aliphatic alcohols and esters **(A,B)**, fatty acids **(C)**, fatty acid ethyl esters **(D)**, and thiols and other sulfur components **(E,F)**. The detection limit is specified for the components for which no detection was possible. The analytes pentanoic acid, dodecanoic acid, ethyl hexanoate, and propane-1-thiol (all not shown) were not detected in any sample. Data are the mean value of biological duplicates ± mean deviation. YE, yeast extract.

Like previously shown, the fermentations with the combination Leu + Han without adding YE differed when using fig syrup or dried fruits. These results (cf. Figure 5) were confirmed by the analyses of the volatile components (Figures 3, 6). That is why further results are compared with those of the previously described trials, in which dried figs were used.

The comparison of the volatile components, displayed in Figure 3 (basis medium) and Figure 6 (modified medium, 0 g/L of YE), in the two main media largely showed similarities. Differences occurred in the amount of thiols and sulfurous components. Methanethiol was twice as high in the modified medium with fig syrup regardless of the additional usage of YE media compared with the medium with dried figs, and the associated component ethanethiol was 10-fold higher, which is displayed in Figures 6E,F, respectively.

The analyses of the volatile components of the products fermented by Leu + Han with the usage of different concentrations of YE and the replacement of dried figs by fig syrup showed a smaller amount of esters and higher aliphatic alcohols, e.g., acetaldehyde and ethyl acetate and can be seen in Figure 6, compared with the former experiments (cf. Figure 3). The formerly solely detected aliphatic alcohol—2-phenylethyl acetate—was under the range of detection without the supplementation of YE, whereas with the addition of YE, half as much was formed in maximum than observed as shown in section “Different Lactic Acid Bacteria–Yeast Combinations for Producing Water Kefir Beverages.” Thereby, the higher the YE concentration, the higher the formed amount was. In contrast to the lower amount of some fermentation by-products, in detail, higher aliphatic alcohols as well as esters (part A and B), the amount of fatty acid ethyl esters was similar or increased. This was associated with the usage of 1 g/L of KAT YE, compared with the former experiments without YE and dried figs, and comparable with the amounts in the unfermented media.

**FIGURE 7:**
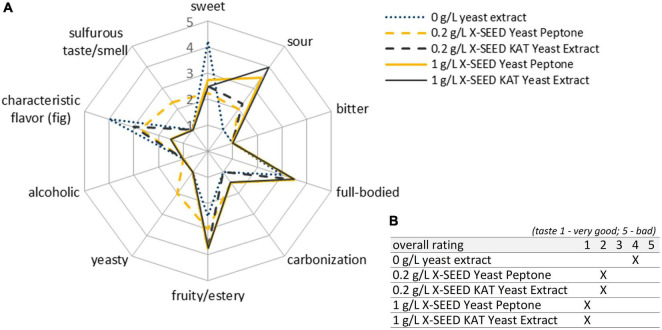
Sensory properties of the products fermented with the microbial combination Leu + Han under the investigation of 0, 0.2, and 1 g/L of two different YEs after 7 days of fermentation. Shown are the mean data of two samples produced in biological duplicates. The rating scale of the descriptive analysis **(A)** ranges from 1 (imperceptible) to 5 (very pronounced). The overall rating of the taste **(B)** ranges from 1 (very good) to 5 (bad taste). YE, yeast extract.

The fatty acid concentration in the products during the supplementation of YE decreased similarly to the product previously described, and no influence of different YEs as well as concentrations was observed. Interestingly, the concentration of butanoic acid of the fermented product without YE was the highest in all experiments at about 24 mg/L. The associated rancid bad taste was clearly identified in the sensory evaluation as well (cf. Figure 7).

Of particular note during these experiments was the presence of sulfurous components, recognizable in detectable volatile components (Figures 3E,F, 6E,F) as well as smell and taste (cf. Figures 4, 7). Although most of the analyzed sulfurous components in the unfermented modified media were concentrated higher than in the basis medium, their amount was reduced by the addition of YE, except for methanethiol, which was in the same range. The concentrations of ethanethiol were 5- to 10-fold lower as well as the formed amount of dimethyl disulfide, which was about 10-fold lower, except for the addition of 0.2 g/L of X-SEED Yeast Peptone. Thereby, the highest amount of dimethyl disulfide was produced during the trials, which was still ∼30% lower than those without YE. The detected concentration of dimethyl trisulfide in the product without YE was similar to the former experiments, and reduced to levels under the range of detection with the addition of YE, except for the addition of 0.2 g/L of X-SEED Yeast Peptone, which led to 3-fold higher concentrations of this. This reduction in sulfurous components was also observed in the sensory analysis of the products and is displayed in Figure 7.

The final products showed improved sensory properties, recognizable in a higher pronounced sourness, more full-bodied products, and more fruitiness as compared with the study without YE in co-cultivation (dotted blue line in Figure 7). Thereby, the expression depended on the applied YEs and their concentration. The supplementation of the tested YEs avoided the production of noticeable sulfurous components at the end of the fermentation except for the usage of 0.2 g/L of X-SEED Peptone Yeast Peptone, as shown in the dashed yellow line in Figure 7. Concluding, the usage of 1 g/L of YE of both X-SEED KAT and X-SEED Peptone showed the best final products after 7 days of fermentation. That is why the next investigations were performed under the supplementation of X-SEED KAT YE with the microorganism combination Leu + Han. Through the fact that no YE off-flavor was detectable in taste and smell, a slightly higher concentration amounting to 1.3 g/L of YE was used in following studies.

### Five-Liters Bioreactor Fermentation With Water Kefir Grains and Defined Co-culture Leu + Han

**FIGURE 8:**
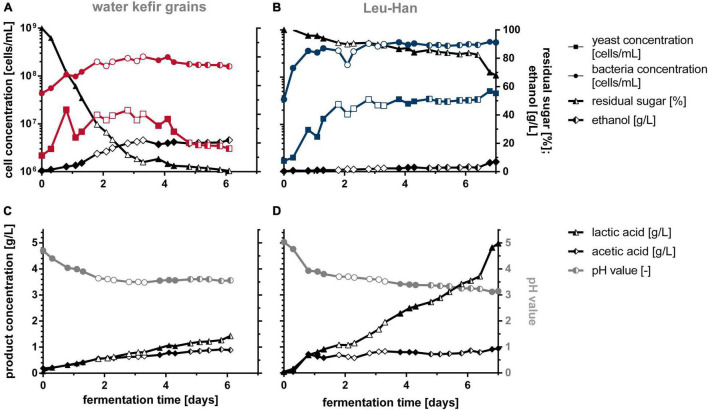
Off-line data of WK fermentations in 5-L bioreactors with grains **(A,C)** and the defined co-culture Leu + Han **(B,D)**. Fermentations were conducted in biological triplicates. Data from identical sampling times are shown as the mean value of a triple (full-filled mark), double (half-filled mark), or single (unfilled mark) sampling. WK, water kefir.

In order to estimate the reproducibility of the WK production by co-cultivation compared with WK grains, fermentations in 5-L bioreactors were performed (Figure 8 and Supplementary Figure 2). For the fermentation, fig syrup was used, and in case of co-cultivation, 1.3 g/L of X-SEED KAT YE was used additionally as tested before. Figure 8 shows the averaged values of triple, double, or single sampling.

It is of particular note that the grain-based fermentations gave desirable sensorially similar products after 3, 6, and 5 days, whereas beverages produced by co-cultures were sensorially similar after 7 days of fermentation each. This was not obvious from the measured data but is of importance for later industrial application. The reached cell concentrations of yeast and bacteria were higher in co-cultivation than during the fermentation with grains (∼3-fold for bacteria and 10-fold for yeast). Incidentally, the yeast concentration decreased after 3 days of fermentation by using grains, which is most probably reasoned by the low stirring rate and the impeded homogenization caused by an increasing grain mass during fermentation. The gray lines in Figure 8 show a similar pH progress in fermentation by using grains and co-cultivation of the two microorganisms until the fourth day of fermentation. Afterward, the pH of the grain fermentation stagnated, whereas the pH in co-culture further decreased to a final value of 3.14.

The usage of grains led to higher ethanol concentrations (23 g/L at 3 days; ∼3 Vol.-%) than the co-cultivation of Leu + Han, accompanied by a nearly complete consumption of the sugar at the fourth day of fermentation, which is comparable with the fermentations in section “Different Lactic Acid Bacteria–Yeast Combinations for Producing Water Kefir Beverages” in 1-L scale. After 6 days of fermentation with co-cultures, the amount of ethanol increased to 3.3 g/L (0.42 Vol.-%), and to a final value of 7 g/L on average after 1 week of fermentation rapidly. When comparing the three fermentations with co-cultures, the sugar consumption as well as the ethanol content in the end deviated (14.8 vs. 2.7 and 3.5 g/L), although staying below 3 g/L until 6 days of fermentation, which is why the mean value of ethanol was that high. The lactic acid concentration reached higher values by co-cultivation than by the fermentation with grains (4.98 vs. 1.33 g/L), which was recognizable in the taste of the final products.

Additional online analyses of the pH, dissolved oxygen, and redox value are illustrated in Supplementary Figure 2. In summary, these data confirm the fermentation processes shown in Figure 8. The essential differences, namely, that the sugar is consumed faster when using grains and proportionally fewer organic acids are formed from it, is confirmed by the online curves. It also illustrates the rapid setting of anaerobic conditions in both approaches. The dissolved oxygen was consumed within the first 2.5 h when applying grains, whereas the usage of Leu + Han resulted in full oxygen consumption after 8 h of fermentation. This was largely due to the activity of the yeasts in the beginning of the fermentation, whereby the defined one only contained one yeast strain.

**FIGURE 9:**
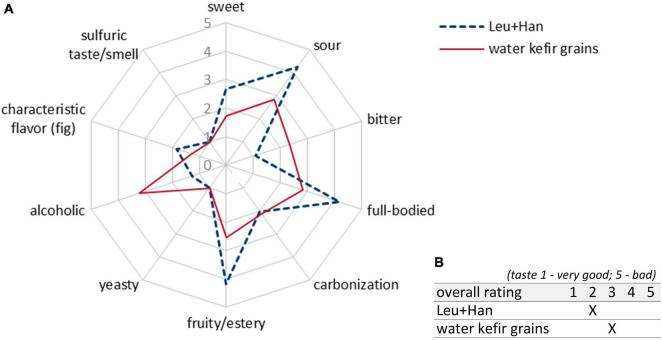
Sensory properties **(A)** and rating **(B)** of products fermented with the combination of Leu + Han as well as WK grains in 5-L bioreactors. Shown are the mean data of biological triplicates at the best time of each single fermentation depending on the best achieved sensory properties: Leu + Han at 7 days of fermentations. Grain trial 1 (f1) at 3 days, trial 2 (f2) at 6 days, and trial 3 (f3) at 5 days of fermentation. The rating scale of the descriptive analysis **(A)** ranges from 1 (imperceptible) to 5 (very pronounced). The overall rating of the taste **(B)** ranges from 1 (very good) to 5 (bad taste). WK, water kefir.

Figure 9 shows the mean scores of sensory properties of biological triplicates at the best time of each single fermentation depending on the best achieved sensory properties as stated above (3, 6, and 5 days for grain fermentations; 7 days for co-culture fermentations). The final products of the fermentation with the co-cultivation of Leu + Han (blue dashed line in Figure 9) showed more desirable sensory properties in line with the study aim of a balanced low-alcoholic beverage than the product produced by the fermentation with grains (red line in Figure 9). Thereby, a higher value of sourness as well as a fruitier taste was observed (Figure 9). Furthermore, the products were more full-bodied and not that much alcoholic than the products fermented by using grains. Further, no off-flavor based on sulfurous taste or smell was observed in any of the products.

## Discussion

The aim of this study was to expand the knowledge about the impact of different microbial consortia representing different degrees of complexity on the characteristics of WK. Therefore, LAB and yeasts isolated from WK grains were used for fermentations as defined starter cultures in co-cultivation against the original grains as a control. The focus of the trial was firstly to reduce the complexity of WK fermentations in order to provide a more controllable and reproducible process and secondly to monitor differences in cell growth, the corresponding metabolization of sugars, production of organic acids, and the perceptible taste in combination with chemical analysis of the microbial volatile components.

### Water Kefir Production With Complex Grains and Defined Starter Cultures

Based on previous internal studies regarding isolated defined strains from traditional WK grains (data not shown), combinations of two different LAB, *L. nagelii* (Liq) and *L. mesenteroides* (Leu), as well as three different yeasts, *H. valbyensis* (Han), *D. bruxellensis* (Dek), and *S. cerevisiae* (Sac), were applied within the present study. Comparative fermentations with six microorganism combinations in co-cultivation as well as kefir grains were performed.

In summary, the fermentations with grains were much faster than in co-cultivation, which could be explained by a high adaptation of the higher number of microorganisms in grains among one another in contrast to only two species that might not be able to fully compensate for all microbial interactions (Figure 1). This is supported by data from Laureys et al. (2021), who also found that WK liquor as a fermentation starter to progress slower as compared with the usage of grains. Metabolic exchanges, which can act as a power source as well as a supply of microbial growth factors, were reported before (Prado et al., 2015), whereby the mutual interactions can appear directly as well as indirectly. In a consortium of several different microbial strains, a possible undersupply of nutrients can be compensated, whereas in co-cultivation, the nutrition exchange is limited to the present microbial strains. That is why the production of WK by using a preferably small number of microbial strains is possible but is less self-regulating and thus requires more process knowledge. However, the conducted fermentations with WK grains resulted in a final product characterized by ethanol levels of around 20 g/L as well as higher amounts of higher alcohols compared with co-culture trials, which could be observed by Laureys and de Vuyst (2014) as well. In line with Chen et al. (2009), who declared that the application of co-culture processes for the WK production could sidestep the intense formation of alcohols, the beverages produced with defined co-cultivations showed significantly lower ethanol formation in the present study. The resulting effects could not be traced back to the performance of one single microorganism in the applied co-cultivations, but rather to microbial interactions.

Combinations including *S. cerevisiae* showed the lowest growth of the investigated LAB in co-cultivation, especially in the case of co-cultivated *L. mesenteroides*, compared with the other applied yeasts, which resulted in the lowest produced amount of lactic acid in association with the lowest observed pH drop (Figures 1C, 2). This resulted in less pronounced WK typical characteristics. In accordance with this, Stadie et al. (2013) observed a poorer growth of *L. nagelii* in co-cultivation with *S. cerevisiae* compared with the co-cultivation with the yeast *Zygotorulaspora florentina* in their investigations of metabolic activity and symbiotic interactions of LAB and yeasts isolated from WK. Similar observations were described by Bechtner et al. (2019). They investigated proteomic analyses of *L. nagelii* in the presence of *S. cerevisiae* isolated from WK. In contrast, the study of Jin et al. (2019) did not demonstrate a negatively affected growth rate of *Lactobacillus plantarum* by the presence of *S. cerevisiae* but a possible inhibition of yeast by *L. plantarum*, which is recognizable in a lower growth rate of the population compared with the single culture of the yeast. It is obvious that a generalization of the microbial interactions during the co-cultivation of a lactic acid bacterium with *S. cerevisiae* as well as other yeast species is not possible and has to be examined individually.

Different gas chromatographic methods were conducted to quantify volatile components in the fermented products. Laureys and de Vuyst (2014) found ethyl acetate, isoamyl acetate, ethyl hexanoate, ethyl octanoate, and ethyl decanoate as the most prevalent volatile components in their investigations of the microbial species diversity, community dynamics, and metabolite kinetics of WK fermentation with the usage of grains. Except for ethyl hexanoate, these components were produced in different amounts during their fermentations with the tested combinations, whereby ethyl acetate was the most prevalent component (Laureys and de Vuyst, 2014). Walsh et al. (2016) pointed out a correlation between *Saccharomyces* spp. and esters’ production during their investigations regarding microbial succession and flavor production in the fermented dairy beverage kefir. In accordance with Walsh et al. (2016), *S. cerevisiae* combined with *L. mesenteroides* led to the highest amount of ethyl acetate in the present study. The usage of *D. bruxellensis* showed a different behavior in the formation of ethyl acetate, whereby in combination with *L. nagelii* no formation and in combination with *L. mesenteroides*, a low formation of ethyl acetate occurred. The presence of higher aliphatic alcohols after 7 days of fermentation was observed, whereby a high variety especially during the usage of grains occurred. The combination of *H. valbyensis* and *L. nagelii* led to the highest detectable amounts of these followed by grains and the usage of *S. cerevisiae*. The combination containing Dek formed a lower amount of higher aliphatic alcohols and no amount of esters in a detectable range. Compared with the other co-cultures, the lowest degradation of fatty acids occurred, which may be attributed to a lower fermentation activity of Dek. In contrast, the associated ethyl esters were present in the highest amount, which led to fruity characteristics of the products. This could be explained by a high noticeable fig taste after 7 days and, consequently, less pronounced sensory properties of these products due to a lower fermentation performance of *D. bruxellensis*. This could be due to slightly lower growth rates of *D. bruxellensis* in the microbial combinations with *L. nagelii* and *L. mesenteroides* compared with the other tested combinations. Former studies including *D. bruxellensis* showed a similar behavior regarding a slow growth rate in the beginning of fermentation (e.g., Abbott et al., 2005), which might promote the LAB growth rate and increase the beginning acidification. Thus, the combinations including LAB and Dek had a specific effect on the fermentation performance favoring LAB growth and acidification. Furthermore, a strong-off flavor, which was describable as “stuffy” and “stale,” was observed in these products. In the wine industry, Dek is well-known for the production of the observed flavors and thus considered as a wine-spoilage yeast. Among other studies, this observation was mentioned by Lambrechts and Pretorius (2019) in their investigations of yeasts’ importance to wine aroma in context of wine contamination with *D. bruxellensis*. Whereas in other contexts the combination of *Dekkera* and LAB was advantageous [e.g., in a commercial alcohol production process described by Passoth et al. (2007)], in the case of the present study, the usage of this yeast in co-culture was not appropriate for WK production.

In contrast to the presence of fruity notes, in all products except for the combinations with *D. bruxellensis*, a sulfurous off-flavor in taste and smell was observed. This was more pronounced when using *L. nagelii* than *L. mesenteroides*, which led to the conclusion that the production of these components might have been influenced by the presence of the lactic acid bacterium in co-culture. Accordingly, this observation was confirmed by the volatile component analysis. The unfermented medium already contained small amounts of methanethiol, ethanethiol, diethyl sulfide, and dimethyl disulfide most probably originating from the figs. In the fermented products, however, high amounts of methanethiol as well as ethanethiol and dimethyl disulfide, ethyl thioacetate, and dimethyl trisulfide occurred, which share the thiol precursor methanethiol (Landaud et al., 2008; Kinzurik et al., 2020). Landaud et al. (2008) described the formation of these thiols due to the degradation of sulfur/carbon bonds of methionine or cysteine derivates during their investigation of the formation of volatile sulfur components and metabolism of methionine and other sulfur components in fermented food. Particularly hydrogen sulfide, methanethiol and ethanethiol as volatile sulfurous components are often sensed as garlic, rotten eggs, onion, and fermented cabbage (Moreira et al., 2002; Landaud et al., 2008). These components have a negative impact on the beverage aroma, when they are present in an amount higher than their perception thresholds. This is especially observed in wine and often is being traced back to enzymatic formation resulting from yeast metabolism (Moreira et al., 2002). The component H_2_S as an undesirable possible by-product during the alcoholic fermentation of *S. cerevisiae* caused by the conversion of cysteine, can further be converted from ethanol to ethanethiol (Landaud et al., 2008), which was in a detectable range during all co-cultivations as well as during the usage of grains. The quantities of the components methanethiol, ethanethiol, dimethyl disulfide, and dimethyl trisulfide were the highest of all tested sulfurous components, whereby the combination of *L. nagelii* and *H. valbyensis* as well as *S. cerevisiae* showed the highest formation.

Irrespective of the occurred off-flavors, the combination of the yeast *H. valbyensis* and the lactic acid bacterium *L. mesenteroides* showed the highest potential in order to develop a water kefir beverage based on the usage of defined microbial strains. This combination in co-cultivation led to the best final product with high-pronounced attributes, such as lactic acidic, full-bodied, fruity, and little carbonized. In this context, the sensory characteristic of the final products played a crucial role in the evaluation of an appropriate combination of microorganisms. Overall, no undesirable microbial interactions were evidenced during this microbial combination, which affected the growth rates as well as microbial metabolisms of these participating microorganisms adversely; thus, this combination was used for further investigations.

### Adaption of the Fermentation Basis Medium by Supplementation of Yeast Extract and Substitution of Dried Figs by Fig Syrup

Regardless of the type of the chosen starter cultures, in addition to a carbon source (mostly raw cane sugar or pure sucrose), most dried fruits (e.g., figs or cranberries) are used for the production of WK. Although the latter serves as a source of amino acids, vitamins, and minerals, the small applied amount of dried fruits compared with the richness in nutrients of fresh fruits leads to a WK medium relatively poor in nutrients (Laureys et al., 2018). One step in the process development in this study aiming for a more defined product was the replacement of dried figs with liquid fig syrup that is well-defined (see Manna-Feigen-Sirup product sheets). Pretests were used to set the concentration in such a way that it corresponded to the previously proven use of dried figs in terms of sensorial and chemical parameters during fermentation, in which 9 mL of fig syrup per liter sugar water was considered as appropriate. The executed fermentations with grains and syrup did not differ to the usage of dried figs in their main performances (Supplementary Figure 1). However, the sole use of sucrose and fig extract in co-cultivation led to a nutritional undersupply of the starter cultures and in particular of the applied lactic acid bacterium (*L. mesenteroides*), which was expressed in a reduced bacterial growth rate and acid formation (see Figure 5; 0 g/L of YE). This led to an unbalanced product with poor sensory ratings, for instance, high sweetness as well as a high-pronounced characteristic fig flavor and low acidity, which were perceived as unpleasant in this combination (Figure 7; 0 g/L of YE). Consequently, in the course of the investigations, two essential factors could be identified that are crucial for the production process of this beverage under defined conditions: efficient nutrient supply and prevention of the formation of sulfurous metabolic products, which can be caused by yeast metabolism, especially in the case of an undersupply of assimilable nitrogen components (Song et al., 2020).

In order to overcome nutritional limitations in co-culture approaches, the supplementation of YE in different concentrations was investigated. Vardjan et al. (2013) described a decreased biochemical activity as well as no growth of various microorganisms, which were separated as pure cultures for milk kefir production, in their studies on the characterization and stability of lactobacilli and yeast microbiota in kefir grains. In order to overcome these issues, they mentioned an improvement by the addition of YE to the growth medium, which was done in the present study as well. The X-SEED KAT YE contains a high content of free amino acids and is rich in B vitamins and minerals, whereas the X-SEED Peptone yeast peptone contains almost 80% of the available amino acids in the form of small peptides. The application of YEs as shown here (Figures 5, 6) promoted growth rates of LAB, which led to higher acidity, and was associated with a higher lactic acid formation and pH drop of the products as compared with fermentations without the supplementation of YE. This was also recognizable in well-balanced sensorial properties of the final products, e.g., a higher acidity and more full-bodied profiles (Figure 7). Thereby, the extent of the improved performances was dependent on the concentration of the applied YE: the higher the concentration, the better the fermentation performance of the LAB as well as the yeast. However, there was no clear preference in either of the yeast derivatives, concluding that *L. mesenteroides* was able to use the nitrogen in the form of free amino acids as well as in the form of peptides equally well. Contrarily, no promotion of the growth rate of *H. valbyensis* by adding YE during the co-cultivation with *L. mesenteroides* was detectable at all.

Laureys et al. (2018) investigated the influence of different fruits or YE/yeast peptone on the WK fermentation process using grains. During their studies, different fermentation characteristics, indicated by formation of ethanol (21.3 g/L using figs; 26.1 g/L applying a mixture of YE and peptone) and lactic acid production (2.83 g/L using figs; 2.06 g/L applying a mixture of YE and peptone) among others were observed (Laureys et al., 2018). Interestingly, within this study, no excessive production of ethanol was observed by the supplementation of YE applying the co-cultivation of *H. valbyensis* and *L. mesenteroides* (e.g., ∼1.2 g/L of ethanol using 1 g/L of X-SEED KAT YE). A lower production of fermentation by-products as well as sulfurous metabolic components was observed, which is most likely based on the yeast metabolism. The concentration of methanethiol was in the same range comparing fermentations without YE and with the usage of dried figs (Figures 3, 6). However, the amount of the associated metabolites, for instance, ethanethiol as well as dimethyl disulfide, has been reduced by about 10-fold (Figures 3, 6). This was recognizable in smell and taste. The final products including YE exhibited no sulfurous aroma in smell and taste, except for the usage of 0.2 g/L of X-SEED Peptone yeast peptone. Accordingly, this product showed the highest amount of thiols and sulfurous components detected via gas chromatographic analysis after fermentation.

Concluding, the addition of at least 1 g/L of YE, especially the applied X-SEED KAT YE, promoted a nutritional supply and might balance a lack of nutrition, which avoids the production of high amounts of sulfurous components when defined starter cultures are used. Furthermore, this effect allowed the replacement of dried figs with fig syrup. These steps led to a more defined process of the WK production.

However, the concentration of YE could be increased in order to decrease the fermentation time and therefore the production process bearing in mind avoiding noticeable yeasty flavor components in smell or taste. The usage of X-SEED KAT YE showed slightly higher lactic acid concentrations (Figure 5E) in the development of a WK-like beverage by co-cultivation consisting of one LAB and yeast, respectively, and thus, further investigations with 1.3 g/L of X-SEED KAT YE were conducted.

### Characterization of Water Kefir Fermentation Based on Grains and Defined Co-cultures in 5-L Bioreactors

The development of a defined process for WK production instead of using complex grains enables the possibility to produce a WK-like beverage by using the co-cultivation of one LAB and yeast. In order to compare the production of WK with grains with the usage of defined co-cultivation under well-monitored and scalable conditions, 5-L bioreactor fermentations were performed in triplicates (see Figure 8 and Supplementary Figure 2). Fermentations performed by a small number of participated microorganism cultures are aimed for because of their higher opportunity to control and guide the process and the properties of the final product (Chen et al., 2009). Further, for a large-scale production, a stable microbial community, which does not change over time, is crucial (Vardjan et al., 2013). Additionally, possible contaminations might be much earlier detectable as compared with a non-defined complex consortium, allowing for early counteractions as well. Although the fermentation with two defined starter cultures is still a complex process, and interactions within the processes are still not fully understood, the fermentation of co-cultures showed a better-defined and more controllable process. This led to a more reproducible production process compared with the fermentation with WK grains, which showed high fluctuation regarding the time when the beverage had the best sensory properties (3, 5, and 6 days; cf. section “Five-Liters Bioreactor Fermentation With Water Kefir Grains and Defined Co-culture Leu + Han”). The usage of WK grains has been a traditional method for many years, which is similar to the milk kefir production. However, the industrial production of WK by using grains is challenging. There are only few opportunities to control the process (Chen et al., 2009; Laureys and de Vuyst, 2017), which leads to a lower reproducibility and might end in high alcoholic products, which were observed in the present study. The fermentations with grains showed a high formation of ethanol during the first 3 days of the fermentation with a final amount of 23 g/L. Laureys and de Vuyst (2014) also noted a high production of ethanol in WK (around 20 g/L), which was produced by using grains. In comparison, with the co-culture, only 0.49 Vol.-% ethanol was produced until the time the beverage was “ready,” which consequently complied with the legislation in the regions of Germany, Austria, and Switzerland, which permit an alcoholic content of 0.5 Vol.-% as a maximum in beverages declared as “alcohol-free” (Das Eidgenössische Department des Innern, 2005). In contrast, the product fermented by Leu + Han showed a significantly higher residual sugar content than did the grain fermentation. As illustrated in Figure 8, ∼80% of the originally inserted amount of sugar (60 g/L) was found after 6 days of fermentation corresponding to ∼48 g/L. The World Health Organization recommends a sugar uptake of free sugars to <10% of total energy intake corresponding to ∼50 g/L of free sugars for an adult having a diet of 2,000 kcal (World Health Organization [WHO], 2015). However, in comparison with common soft drinks, such as Coca-Cola (14 FO bottle; 111 g/L of sugars), Pepsi (20 FO bottle; 118 g/L of sugars), or Red Bull (8.4 FO can; 109 g/L of sugars) (Ventura et al., 2011), the WK produced by co-culturing within the present study would be considered as a low-sugar-containing beverage according to the classification of Bandy et al. (2020). With regard to further potential health benefits associated with fermented drinks in general, the herein presented product would be competitive on the beverage market.

Summarizing, the WK beverage gained by co-cultivation with *L. mesenteroides* and *H. valbyensis* differed in the determined cell count, acidity, fermentation duration, ethanol content, and consequently its sensory profile as compared with the grain-based WK. Wang et al. (2016) encapsulated yeast, LAB, and acetic acid bacteria in liquid core capsules and achieved a volatile aroma profile close to that of the grain-based product in milk kefir. WK produced by Laureys and de Vuyst (2014) by using grains contained 4.9 g/L of lactic acid and 1 g/L of acetic acid, which is in accordance with the metabolite concentrations of the developed fermented beverage without using grains in this study. However, two different products were gained in the present study, whereby both products showed typical attributes of WK. In addition, the co-cultivated products performed slightly better in sensory terms than those made with grains (cf. Figure 9) regarding a low alcoholic balanced sour and fruity drink. These first results are therefore very promising for further beverage developments to be based on. Under this aspect, the studies carried out here can be understood as a piece of the puzzle in order to establish knowledge-based production processes for complex fermentation products such as WK, so that these are more controllable and reproducible, especially for industrial production. The first studies were conducted by Stadie et al. (2013) as well as Bechtner et al. (2019), which made symbiotic interactions of co-cultures for WK production subjects of discussion.

## Conclusion

The work presented herein described steps from a traditionally produced process to a defined water kefir fermentation process applying a co-culture of only one LAB and one yeast, respectively. It could be shown that the main characteristics of WK—a fruity, aromatic, and acidic beverage made by fermentation of characteristic strains—were achieved by the use of two microbes *L. mesenteroides* and *H. valbyensis*, although the chemical analyses revealed differences compared with those of the undefined grain origin. However, the defined consortium outperformed the grains, as considerably lower levels of ethanol were formed. These results proved the possibility to reduce the complexity of the fermentation process by keeping the aimed product characteristics at the same time. The here-examined defined microbial consortia for the production of WK caused different product characteristics, opening up further space for detailed research and in which remaining challenges, e.g., a possible optimization of the fermentation time, should be addressed. Especially, the usage of one LAB and one yeast each is a promising approach in order to get deeper insights into microbial interactions during fermentation and simultaneously avoiding blurring effects due to an uncontrollable number of associated actors.

## Data Availability Statement

The raw data supporting the conclusions of this article will be made available by the authors, without undue reservation.

## Author Contributions

AT, MSc, ML, and SK conducted the experiments. NR was responsible for the aroma analyses. MSe and SK performed the data analyses. MSe, MSc, and SK drafted the manuscript. ML reviewed and contributed to the structure and content of the manuscript. All authors contributed to the article and approved the submitted version.

## Conflict of Interest

The authors declare that the research was conducted in the absence of any commercial or financial relationships that could be construed as a potential conflict of interest. The reviewer SJ declared a shared affiliation with one of the authors MSc, to the handling editor at time of review.

## Publisher’s Note

All claims expressed in this article are solely those of the authors and do not necessarily represent those of their affiliated organizations, or those of the publisher, the editors and the reviewers. Any product that may be evaluated in this article, or claim that may be made by its manufacturer, is not guaranteed or endorsed by the publisher.
