# Supplementary material for: Tradition as a Stepping Stone for a Microbial Defined Water Kefir Fermentation Process: Insights in Cell Growth, Bioflavoring, and Sensory Perception
Source: Front Microbiol. 2021 Nov 4;12:732019. doi: 10.3389/fmicb.2021.732019 (PMC9336596; doi:10.3389/fmicb.2021.732019)
Supplement: Supplementary file 1 [file Data_Sheet_1.docx]

Supplementary Material

# Supplementary Figures and Tables

## Supplementary Figures


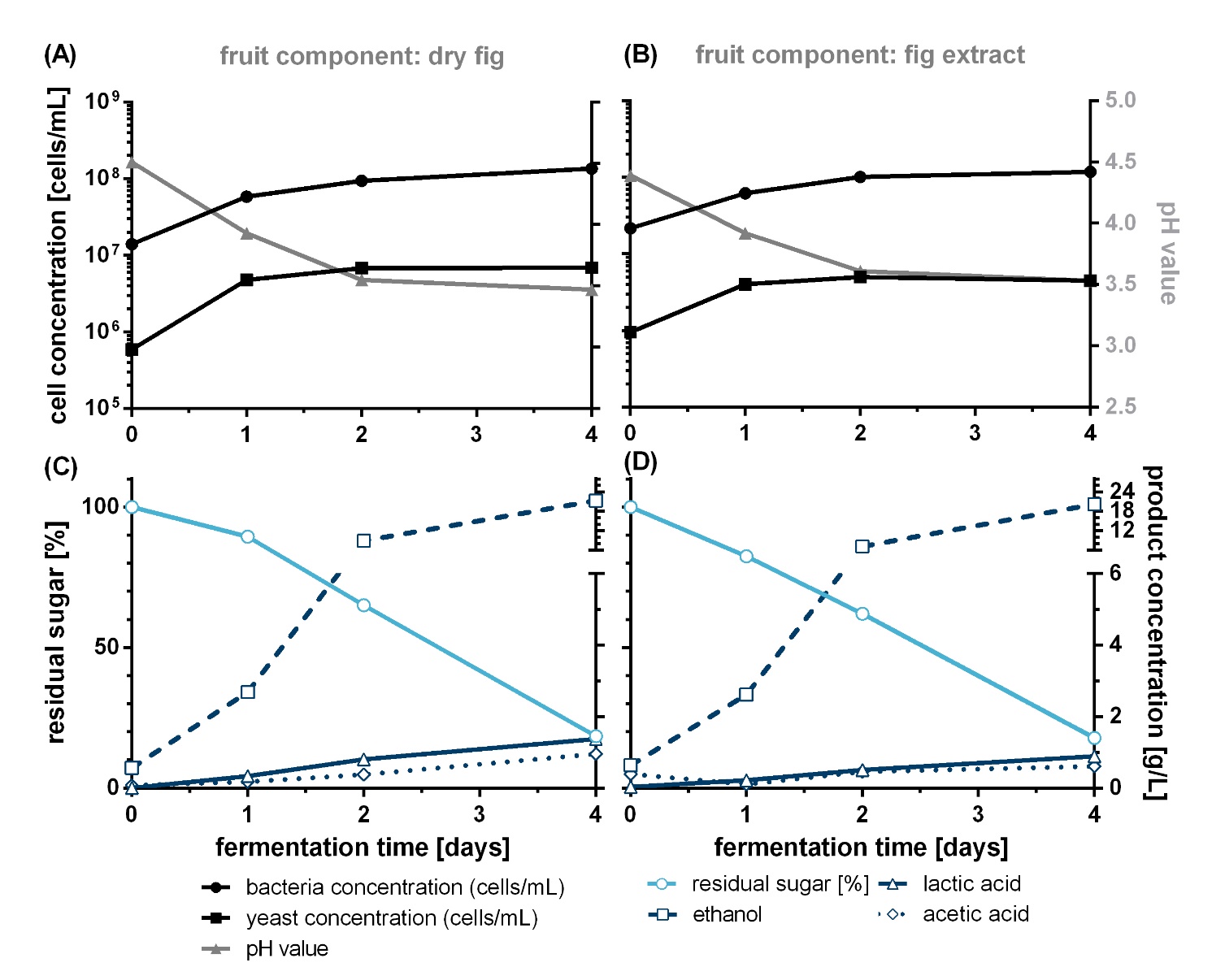


**Supplementary Figure 1.** Course of cell concentrations and pH value (A+B), as well as the produced metabolites and consumed sugar (C+D) during the WK fermentations applying grains with dried figs (A+C) and fig concentrate (B+D).


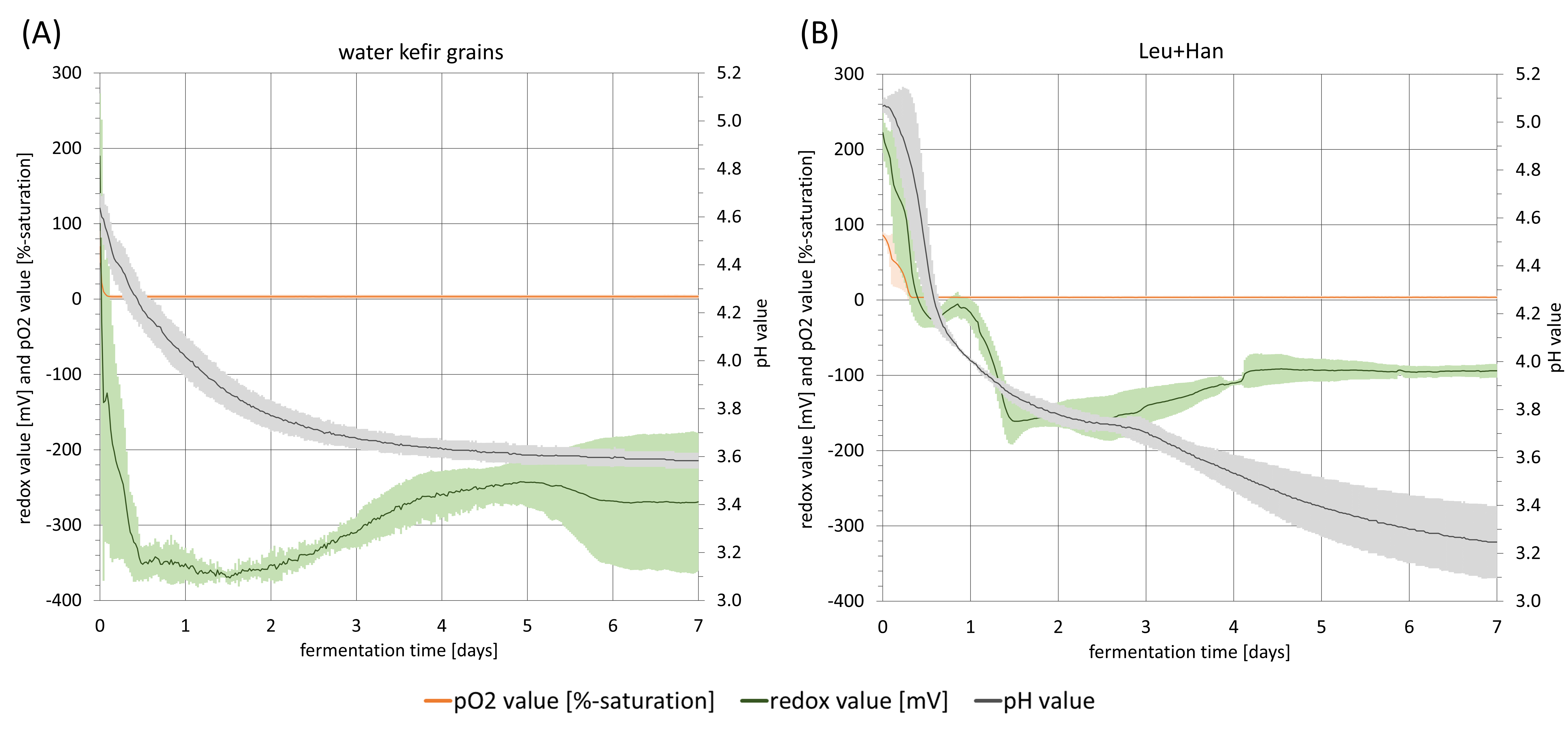


Supplementary Figure 2: Course of the on-line redox-, pO_2_- and pH-value of WK fermentations in 5 L bioreactors with grains (A) and a defined co-culture of *Leuconostoc mesenteroides* and *Hanseniaspora valbyensis* (B). Fermentations were conducted in biological triplicates. Data represent the mean value of triplicates and the corresponding standard deviation.
